# Protective Effect of Seasonal Influenza Vaccination in Elderly Individuals with Disability in Taiwan: A Propensity Score–Matched, Nationwide, Population-Based Cohort Study

**DOI:** 10.3390/vaccines8010140

**Published:** 2020-03-22

**Authors:** Yu-Chia Chang, Huang Yu-Tung, Long-Sheng Chen, Ho-Jui Tung, Kuang-Hua Huang, Ernawaty Ernawaty, Szu-Yuan Wu

**Affiliations:** 1 Department of Healthcare Administration, College of Medical and Health Science, Asia University, Taichung 41354, Taiwan; ycchang@asia.edu.tw; 2 Department of Medical Research, China Medical University, Taichung 40402, Taiwan; 3 Center for Big Data Analytics and Statistics, Chang Gung Memorial Hospital, Taoyuan 333, Taiwan; anton.huang@gmail.com; 4 Surveillance, Research and Health Education Division, Health Promotion Administration, Ministry of Health and Welfare, Taipei 10341, Taiwan; lschen@hpa.gov.tw; 5 Department of Health Policy and Community Health, JPH College of Public Health, Georgia Southern University, Statesboro, GA 30458, USA; htung@georgiasouthern.edu; 6 Department of Health Services Administration, China Medical University, Taichung 40402, Taiwan; khhuang@mail.cmu.edu.tw; 7 Department of Health Policy and Administration, Faculty of Public Health, Universitas Airlangga, Surabaya 60115, Indonesia; ernawaty@fkm.unair.ac.id; 8 Division of Radiation Oncology, Lo-Hsu Medical Foundation, Lotung Poh-Ai Hospital, Yilan 265, Taiwan; 9 Big Data Center, Lo-Hsu Medical Foundation, Lotung Poh-Ai Hospital, Yilan 265, Taiwan; 10 Department of Food Nutrition and Health Biotechnology, College of Medical and Health Science, Asia University, Taichung 41354, Taiwan

**Keywords:** influenza vaccination, elderly, disability, severity, mortality

## Abstract

This is the first and largest population-based cohort study to demonstrate that influenza vaccination reduced all-cause mortality and influenza-related hospitalization in elderly individuals with a disability. Purpose: To estimate the protective effect of influenza vaccination in elderly individuals with a disability by conducting a propensity score-matched (PSM), nationwide, population-based cohort study. Methods: Data from Taiwan’s National Health Insurance Research Database were used in this study. Generalized estimating equations (GEEs) were used to compare outcomes between the vaccinated and unvaccinated cohorts. The GEE logit was used to estimate the relative risks of death and hospitalization after influenza vaccination. Adjusted odds ratios (aORs) were used to estimate relative risk. Results: The matching process yielded a final cohort of 272 896 elderly individuals with a disability (136 448 individuals in each cohort). In multivariate GEE analyses, aOR (vaccinated vs. unvaccinated) and 95% confidence interval (CI) of death were 0.70 (0.68–0.72). The aORs (95% CIs) of hospitalization for influenza and pneumonia, respiratory diseases, respiratory failure, heart disease, hemorrhagic stroke, and ischemic stroke were 0.98 (0.95–1.01), 0.96 (0.94–0.99), 0.85 (0.82–0.89), 0.96 (0.93–0.99), 0.85 (0.75–0.97), and 0.89 (0.84–0.95), respectively. The length of stay and medical expenditure exhibited greater reduction in vaccinated elderly individuals with a severe and very severe disability than in unvaccinated elderly individuals. Conclusions: Influenza vaccination reduced all-cause mortality, influenza-related hospitalization, length of stay, and medical expenditure in elderly individuals with a disability. The decrease in the length of stay and medical expenditure because of influenza vaccination was proportional to the severity of disability.

## 1. Introduction

Social Security Administration defines disability as the inability to engage in any substantial, gainful activity because of medically determinable physical or mental impairments, which can be expected to result in death or have lasted or can be expected to last for a continuous period of not less than 12 months [1]. Elderly individuals or elderly individuals with a disability, particularly those who require assistance and those who are residents of long-term care facilities, might be at a high risk of influenza during an influenza pandemic because of disrupted care or exposure to influenza from their caregivers [2,3]. Thus far, strong evidence to prove that elderly individuals with a disability are a susceptible population and have a high risk of influenza is not available; furthermore, no study has shown the beneficial effects of influenza vaccination in elderly individuals with a disability in terms of survival, influenza-related hospitalization, length of stay (LOS), or medical cost. Therefore, in this study, we examined the effects of influenza vaccination in elderly individuals with a disability and evaluated the effects of influenza vaccination in elderly individuals with varying severity of disability.

Although annual influenza vaccination is recommended for all individuals aged ≥6 months, when the supply of influenza vaccines is limited, individuals at a high risk of complications, people in households of high-risk individuals, and caregivers of high-risk individuals should be the highest-priority recipients [4,5,6]. Highest-priority individuals who are at a high risk of influenza complications include those at the extremes of the age spectrum, pregnant women, immunocompromised hosts, and individuals with specific chronic diseases [4,5,6]. The United States Advisory Committee on Immunization Practices recommends that when influenza vaccine supply is limited, vaccination efforts should be focused on individuals who are 6–59 months or ≥50 years old; have chronic pulmonary disease, cardiovascular diseases, renal diseases, hepatic diseases, hematological diseases, metabolic diseases, or neurological disorders; are immunocompromised or pregnant during the influenza season; six months to 18 years old and receiving aspirin- or salicylate-containing medication; residents of nursing homes or other long-term care facilities; and have extreme obesity with a body mass index ≥40 [4]. Although data are not available to strongly indicate that elderly individuals with a disability are at risk of severe or complicated influenza, influenza might be a risk factor for secondary bacterial pneumonia or influenza-related respiratory diseases, respiratory failure, heart disease, hemorrhagic stroke, or ischemic stroke, which can be severe in such individuals [3].

To resolve the question of the value of influenza vaccination in elderly individuals with a disability, we conducted a propensity score-matched (PSM), nationwide, population-based cohort study.

## 2. Individuals and Methods

### 2.1. Materials and Methods

#### 2.1.1. Data Source

The present study was a population-based, retrospective cohort study. Data used in the analysis were obtained from Taiwan’s National Health Insurance Research Database (NHIRD). Taiwan launched the National Health Insurance (NHI) in 1995; it is a single-payer and mandatory enrollment program. More than 99.9% of the citizens and legal residents of Taiwan are covered by the NHI, and this large computerized database contains information on enrollees’ outpatient visits, hospitalizations, prescriptions, medical procedures, and related diagnoses. Detailed information of the NHIRD is previously reported [7]. Since 2001, the Taiwan government has implemented an annual free influenza vaccination policy for all people aged 65 years and older.

For retrieving medical records of individuals with a disability, we linked the NHIRD to the Disabled Population Profile. In Taiwan, individuals with a disability must register for claiming disability benefits, and their information is maintained in the Disabled Population Profile. By linking the datasets, we identified NHI enrollees with disabilities, and enrollees aged ≥65 years in 2014 were considered for inclusion in the current study. However, the NHI elderly enrollees with disabilities were excluded if they died before the end of 2014; received vaccination but not during the free influenza vaccination period (between October 1 and December 31, 2014); and received more than one shot of influenza vaccine during the study and follow-up periods (between 1 January 2014 and 30 September 2015) to avoid cases with coding errors. A total of 394 490 elderly individuals with a disability were identified for the present study.

Data related to the identification of individuals are encrypted before enrollee data are released to the researchers; consequently, individual privacy is protected. The study was approved by the Chang Gung Medical Foundation Institutional Review Board (IRB No. 201900240B0).

#### 2.1.2. Outcome Measures

Four outcome measures were used to evaluate the protective effect of seasonal influenza vaccination, namely all-cause mortality, influenza-related hospitalization, LOS, and inpatient expenditure during the influenza season. All-cause mortality was determined by linking the NHIRD and the Cause of Death Registry system. Using codes from the International Classification of Diseases, Ninth Revision, and Clinical Modifications (ICD-9-CM), we identified information on enrollees’ hospitalizations, LOS, and inpatient expenditures because of influenza-related complications such as pneumonia (ICD-9-CM codes 480-487), respiratory diseases (ICD-9-CM codes 460-466, 480-487, 490-496, and 500-518), respiratory failure (ICD-9-CM codes 518.81-518.84 or 799.1), heart diseases (ICD-9-CM codes 410-429), hemorrhagic stroke (ICD-9-CM 430, 431, or 432), and ischemic stroke (ICD-9-CM 433 or 434) [8,9] during the studied influenza season. LOS was measured as days hospitalized in facilities providing acute and chronic care. Inpatient expenditures were defined as the sum of inpatient medical expenses, including physician or nursing fees, fees for surgery or procedures, medication fees, fees for laboratory examination or tests, ward fees, copayment, and other miscellaneous fees [9].

The effect of influenza vaccination was evaluated using outcome data collected when influenza-like illness (ILI) activity peaked [9,10] and the data used were the influenza-surveillance data from the Centers for Disease Control (CDC) in Taiwan. The flu season of 2014–2015 was milder but longer than the previous five influenza seasons. According to the surveillance data, ILI activity began to increase at the beginning of January 2015, peaked in June 2015, and plateaued at the end of September 2015 [11]. Therefore, the influenza season, in the present study, was between January and September 2015.

#### 2.1.3. Measures of Predictors

The main independent variable, namely receipt of influenza vaccination by included elderly individuals with a disability, was determined using examining the NHIRD claims data (confirmed using drug codes) between October 1, 2014, and December 31, 2014, when the seasonal influenza vaccines were provided for free to people aged ≥65 years in Taiwan. This variable was dichotomized and coded as 1 (vaccinated) and 0 (unvaccinated).

Other baseline covariates were also incorporated in this analysis, including severity of disability, category of disability, sex, age, premium-based monthly salary, urbanization level, the Charlson comorbidity index (CCI) score, catastrophic illness status, long-term care facility residence, and health care utilization. Information on the severity of disability and the category of disability was obtained from the Disabled Population Profile. The severity of disability was classified into four levels, namely mild, moderate, severe, and extremely severe. Eighteen disability categories are listed in the Disabled Population Profile. The number of cases in some categories was extremely small; therefore, in the present study, only the top seven disability categories, namely physical disability, hearing impairment, major organ malfunction, multiple disabilities, dementia, visual impairment, and psychiatric disorders were included, and the remaining 3% of individuals were classified into a category named “other.” According to their ages, elderly individuals were divided into the following four cohorts: 65–69, 70–74, 75–79, and ≥80 years. According to their premium-based monthly salary, individuals were divided into the following categories: ≤NTD19 273, NTD19 274-NTD22 800; NTD22 801-NTD45 800; and ≥NTD45 801. The urbanization level of the residential area was stratified into three levels, namely urban, suburban, and rural. The CCI scores were categorized as 0, 1–2, 3–4, and ≥5. Health care utilization included the number of outpatient visits, any hospitalization, and health examinations during the preceding nine months (prior to October 2014). The number of outpatient department visits during the preceding nine months was stratified according to the median visits of all elderly individuals (aged ≥65 years) in Taiwan (<18 visits vs. ≥18 visits).

#### 2.1.4. Statistical Analysis

Dissimilar distributions of baseline characteristics were observed in the vaccinated and unvaccinated cohorts; to increase the efficiency of comparisons, propensity score matching was used to reduce confounding and selection bias (World Health Organization, 2018). A multivariate logistic regression model that included sex, age, CCI score, premium-based monthly salary, and urbanization level of the residential area was used to obtain propensity scores for the probabilities of being in the vaccinated and unvaccinated cohorts. Next, we used the caliper matching method (also known as the greedy algorithm) with one-on-one matching to obtain pair-matched cases on vaccination status [12].

Bivariate comparisons of the covariates and influenza status were conducted using the chi-square test. Generalized estimating equations (GEEs) were used to compare measured health outcomes between the vaccinated and unvaccinated cohorts. The GEE with logit-link and binomial distribution was used to estimate adjusted odds ratios (aORs) of receiving influenza vaccination for two outcome measures (death and hospitalization). Poisson distribution was used to evaluate LOS. In addition, a GEE model with a log-link and gamma distribution was incorporated, because health care expenditure was non-normally distributed and right-skewed. Furthermore, this study used stratified analyses to explore the effectiveness of influenza vaccination in elderly individuals with different severities of disability. All analyses were conducted using SAS version 9.4 (SAS Institute, Inc., Cary, North Carolina, USA). A two-tailed *P* value of.05 was considered statistically significant.

## 3. Results

The matching process yielded a final cohort of 272 896 elderly individuals with a disability (136 448 individuals each cohort) eligible for the study; their characteristics are summarized in Supplementary Table S1. The vaccinated and unvaccinated cohorts were comparable in terms of sex, age, premium salary, urbanization level, CCI score, and catastrophic illness status. However, the unvaccinated cohort had higher numbers of elderly individuals with mild and moderate disabilities, and they also had a higher number of hospital admissions in the preceding nine months, when comparing to the vaccinated cohort. On the other hand, the vaccinated cohort had higher numbers of individuals living in long-term care facilities, and they also used more outpatient visits and preventive care service in the preceding nine months, when comparing to the unvaccinated cohort. Moreover, the numbers of individuals with hearing impairment, failure of vital organs, multiple disabilities, dementia, or psychological disabilities were higher in the vaccinated cohort than those in the unvaccinated cohort, and the number of individuals with a physical disability was higher in the unvaccinated cohort than those in the vaccinated cohort (Supplementary Table S1).

Our GEE analysis revealed that influenza vaccine administration was a significant factor of all-cause mortality (aOR = 0.70, *p* < 0.001), after controlling for disability severity, disability type, age, sex, premium salary, urbanization level, CCI score, catastrophic illness status, long-term care facility residence, outpatient utilization, hospital admission, and preventive care service utilization. The vaccinated were less likely to die, when compared to the unvaccinated. Elderly individuals with disability in the vaccinated cohort had significantly lower risks of hospitalization for respiratory diseases, respiratory failure, heart disease, hemorrhagic stroke, and ischemic stroke than did those in the unvaccinated cohort, the aORs (95% CIs) were 0.96 (0.94–0.99), 0.85 (0.82–0.89), 0.96 (0.93–0.99), 0.85 (0.75–0.97), 0.89 (0.84–0.95), and 0.96 (0.94–0.98), respectively (Table 1). 

In Table 2, adjusted GEE analyses indicated that individuals in the vaccinated cohort had shorter LOS and lowered medical expenditure for influenza-related complications than those in the unvaccinated cohort. Significant reduction of LOS (ranging from 8% to 14%) for influenza and pneumonia, respiratory diseases, respiratory failure, heart disease, hemorrhagic stroke, and ischemic stroke were seen among the vaccinated cohort. The reductions in medical expenditure for influenza and pneumonia, respiratory diseases, respiratory failure, heart disease, hemorrhagic stroke, and ischemic stroke among the vaccinated cohort were also significant (ranging from 7% to 19%), comparing to the unvaccinated cohort.

After stratified analysis of the risk of death in elderly individuals with different severities of disability in the vaccinated and unvaccinated cohorts, multivariable GEE analyses showed that the all-cause mortality was lower in the vaccinated cohort than in the unvaccinated cohort. The aORs (vaccinated vs. unvaccinated) derived for elderly individuals with mild, moderate, severe, and very severe disability were 0.67 (0.64–0.71), 0.70 (0.66–0.73), 0.73 (0.68–0.77), and 0.74 (0.70–0.79), respectively (Table 3). Table 4 shows that the aORs of LOS and medical expenditure for influenza and pneumonia, respiratory diseases, and respiratory failure were reduced in the vaccinated cohort than in the unvaccinated cohort, irrespective of the severity of the disability. The reduction (vaccinated vs. unvaccinated) of LOS and medical expenditure exhibited a greater reduction in elderly individuals with a severe and very severe disability. However, aORs (vaccinated vs. unvaccinated) of all-cause mortality exhibited a higher reduction in elderly individuals with a mild and moderate disability (Table 3).

## 4. Discussion

Supplementary Table S1 shows that PSM was conducted appropriately, and no differences were observed between the vaccinated and unvaccinated cohorts in terms of sex [13], age [13], premium salary [14], urbanization level [14], CCI score [15], and catastrophic illnesses status [16], which were associated with the all-cause mortality and influenza-related hospitalization and complications. In addition, we also used multivariable GEE analyses for adjustment of long-term care facility residence [17,18], outpatient healthcare utilization [19,20], hospital admission [21,22], and preventive care service utilization [23], which were associated with the prevalence of influenza vaccination administration [3]. In our analysis, we considered that all covariates might be associated with the primary endpoint or prevalence of influenza vaccine. Our results proved the effects of influenza vaccination in reducing the risk of death, hospitalization for respiratory diseases, respiratory failure, heart disease, hemorrhagic stroke, or ischemic stroke in elderly individuals with a disability (Table 1). In addition, influenza vaccine administration in elderly individuals with a disability reduced LOS and medical expenditure for influenza-related complications (Table 2). Moreover, the decrease in the length of stay and medical cost because of influenza vaccination was proportional to the severity of disability (Table 4). Our findings were valid and reliable; the reduction in all-cause mortality and influenza-related hospitalizations by influenza vaccines in elderly individuals with a disability was observed after appropriate PSM and GEE analyses.

To our knowledge, the present study is the first population-based study to demonstrate that the protective effect of influenza vaccine in reducing all-cause mortality, influenza-related hospitalization, LOS, and medical expenditure in elderly individuals with disability. Medical expenditure of individuals with influenza, including those with influenza-related complications such as pneumonia [24], respiratory diseases [25], respiratory failure [26,27], heart disease [28,29,30], hemorrhagic stroke [31], and ischemic stroke [30], has often led to higher spending. In our study, we found that influenza vaccination was associated with reductions in all-cause mortality, influenza-related hospitalization, LOS, and medical costs decreased among elderly individuals with a disability (Table 1 and Table 2). These findings imply that governments should encourage elderly individuals with a disability to receive influenza vaccination regularly because elderly individuals with a disability are highly susceptible to influenza infection and influenza-related complications. Influenza vaccination is necessary and considerably reduces the medical expenditure of the government on the aforementioned population.

Notably, thus far, studies have not compared the association between influenza vaccination and different severities of disability in elderly individuals. Our study is the first study to demonstrate that influenza vaccination reduced all-cause mortality, irrespective of the severity of disability in elderly individuals. In addition, aORs in all-cause mortality exhibited greater reduction in elderly individuals with a mild and moderate disability than in elderly individuals with severe and very severe disability (vaccinated vs. unvaccinated) (Table 3). In the vaccinated cohort, elderly individuals with amild disability exhibited lower aORs of all-cause mortality than those with severe and very severe disability; the observation may be due to the presence of other competitive mortality in individuals with severe and very severe disability elderly rather than influenza-related mortality. Thus, the survival benefits of the influenza vaccine were reduced by other competitive mortality in individuals with a severe and very severe disability.

However, the influenza-related LOS and medical expenditure for influenza and pneumonia, respiratory disease, and respiratory failure were respectively shorter and lower in the vaccinated cohort in proportion to the severity of the disability. The LOS and medical expenditure (vaccinated vs. unvaccinated) exhibited greater reduction among elderly individuals with a severe and very severe disability than among those with mild and moderate disability (Table 4). The findings of this study also proved the necessity of influenza vaccination in elderly individuals with disability, particularly those with severe and very severe disability. Elderly individuals with a severe disability might be highly susceptible to influenza and may develop highly serious complications that increase influenza-related LOS and medical expenditure for influenza and pneumonia, respiratory disease, and respiratory failure. Our results provided evidence for policymakers that influenza vaccination should be a prevention priority for elderly individuals with a disability, particularly those with a severe and very severe disability.

This study has some limitations. First, the toxicity induced by the influenza vaccine could not be determined. However, demonstrating an improvement in survival after influenza vaccination in elderly individuals in randomized trials is difficult because mortality is a rare endpoint [32]. Thus far, most governments worldwide have established that the advantages of influenza vaccination administration outweigh its disadvantages. Second, because all elderly individuals with disability were enrolled from an Asian population, the corresponding ethnic susceptibility remains unclear; hence, our results should be cautiously extrapolated to non-Asian populations. Third, the diagnoses of all comorbid conditions were based on ICD-9-CM codes. The Ministry of Health and Welfare in Taiwan randomly reviews charts and interviews individuals to verify the accuracy of diagnoses, and hospitals with outlier chargers or practices may be audited and subsequently be heavily penalized if malpractice or discrepancies are identified. In addition, the quality and precision of ICD-9-CM codes in Taiwan have been verified and demonstrated by previous studies [33,34]. Therefore, we believe the conclusions of this study are reliable. Finally, the NHIRD does not contain information on dietary habits, socioeconomic status, or body mass index, and all of these factors are risk factors for mortality. However, considering the magnitude and statistical significance of the observed effects in this study, these limitations are unlikely to have affected the conclusions.

## 5. Conclusions

Influenza vaccination reduces all-cause mortality, influenza-related hospitalization, LOS, and medical expenditure in elderly individuals with a disability. The reduction in LOS and medical expenditure by influenza vaccination is proportional to the severity of the disability.

## Figures and Tables

**Table 1:** Comparison of risks of outcomes of interest between the vaccinated and unvaccinated cohorts.

Variables	Total		Without IV		With IV		With IV vs. without IV (Ref.)							
	(*N* = 272,896)		(*N* = 136,448)		(*N* = 136,448)		cOR	95% CI		*p*-Value	aOR^#^	95% CI		*p*-Value
	Event	Incident (‰)	Event	Incident (‰)	Event	Incident (‰)								
Death	24,368	89.29	13 918	102.00	10,450	76.59	0.72	0.70	0.74	<0.001	0.70	0.68	0.72	<0.001
Hospitalization														
Influenza and pneumonia	29,556	108.30	14,296	104.77	15,260	111.84	1.08	1.05	1.11	<0.001	0.98	0.95	1.01	0.111
Respiratory diseases	41,131	150.72	20,228	148.25	20,903	153.19	1.04	1.02	1.06	<0.001	0.96	0.94	0.99	0.004
Respiratory failure	12,636	46.30	6589	48.29	6047	44.32	0.91	0.88	0.95	<0.001	0.85	0.82	0.89	<0.001
Heart disease	24,589	90.10	12,422	91.04	12,167	89.17	0.98	0.95	1.00	0.085	0.96	0.93	0.99	0.004
Hemorrhagic stroke	1103	4.04	605	4.43	498	3.65	0.82	0.73	0.93	0.001	0.85	0.75	0.97	0.014
Ischemic stroke	4249	15.57	2291	16.79	1958	14.35	0.85	0.80	0.91	<0.001	0.89	0.84	0.95	0.001
Any one of above disease	53,622	196.49	26,631	195.17	26,991	197.81	1.02	1.00	1.04	0.075	0.96	0.94	0.98	<0.001

Abbreviations: IV, Influenza vaccination; ref., reference group; cOR, crude odds ratio; aOR, adjusted odds ratio; CI, confidence interval ^#^ All models were analyzed via the generalized estimating equation. Extraneous factors adjusted in the model contained disability severity, type of disability, age, sex, premium salary, urbanization level, CCI score, catastrophic illness status, long-term care facility residents, outpatient utilization, hospital admission, and preventive care service utilization.

**Table 2:** Comparison of the length of stay and medical expenditure between the vaccinated and unvaccinated cohorts.

Variables	*n*	Length of Stay						Medical Expenditure					
		Mean	SD	Exp (b)	95% CI		*p*-Value	Mean	SD	Exp (b)	95% CI		*p*-Value
Influenza and pneumonia													
Without IV (ref.)	14,296	24.29	32.10					152,071	223,199				
With IV	15,260	22.61	28.55	0.91	0.89	0.94	<0.001	133,165	186,104	0.89	0.86	0.92	<0.001
Respiratory diseases													
Without IV (ref.)	20,228	27.55	41.67					173,926	275,804				
With IV	20,903	24.62	35.52	0.89	0.86	0.91	<0.001	148,062	229,082	0.87	0.84	0.90	<0.001
Respiratory failure													
Without IV (ref.)	6589	38.27	58.44					281,394	367,214				
With IV	6047	33.04	50.71	0.89	0.84	0.94	<0.001	243,127	314,442	0.91	0.87	0.95	<0.001
Heart disease													
Without IV (ref.)	12,422	17.05	26.27					128,819	192,189				
With IV	12,167	15.39	22.19	0.92	0.88	0.95	<0.001	116,959	164,269	0.93	0.90	0.97	<0.001
Hemorrhagic stroke													
Without IV (ref.)	605	25.06	34.58					201,769	355,534				
With IV	498	21.19	26.06	0.86	0.75	0.99	0.046	156,093	202,156	0.81	0.69	0.96	0.012
Ischemic stroke													
Without IV (ref.)	2291	18.72	23.48					113,567	154,690				
With IV	1958	16.55	20.68	0.90	0.84	0.97	0.007	101,478	145,837	0.91	0.84	0.99	0.033
Any one of above disease													
Without IV (ref.)	26,631	59.35	111.85					387,788	731,190				
With IV	26,991	53.93	98.12	0.89	0.86	0.91	<0.001	338,457	618,718	0.88	0.85	0.91	<0.001

Abbreviations: IV, Influenza vaccination; ref., reference group; SD, standard deviation; CI, confidence interval; ref., reference group. All models were analyzed via the generalized estimating equation. Extraneous factors adjusted in the model contained disability severity, type of disability, age, sex, premium salary, urbanization level, catastrophic illness status, long-term care facility residents, outpatient utilization, hospital admission, and preventive care service utilization.

**Table 3:** Stratified analysis to compare the risk of death in individuals with different severities of disability between the vaccinated and unvaccinated cohorts.

Disability Severity	Event	Incident (‰)	aOR^#^	95% CI			*p*-value
Mild	6061	57.12	0.67	0.64	-	0.71	<0.001
Moderate	6909	82.42	0.70	0.66	-	0.73	<0.001
Severe	5848	125.19	0.73	0.68	-	0.77	<0.001
Very severe	5550	153.15	0.74	0.70	-	0.79	<0.001

Note: aOR, adjusted odds ratio; CI, confidence interval. ^#^ All models were analyzed via the generalized estimating equation. Extraneous factors adjusted in the model contained type of disability, age, sex, premium salary, urbanization level, CCI score, catastrophic illness status, long-term care facility residents, outpatient utilization, hospital admission, and preventive care service utilization.

**Table 4:** Stratified analysis to compare the length of stay and medical expenditure for influenza-related complications of individuals with different severities of disability between the vaccinated and unvaccinated cohorts.

	Length of Stay			Medical Expenditure				
Disability Severity	Exp (b)	95% CI		*p*-Value	Exp (b)	95% CI		*p*-Value
**Influenza and Pneumonia**								
Mild	0.95	0.94	0.96	<0.001	0.92	0.87	0.97	0.001
Moderate	0.94	0.93	0.95	<0.001	0.90	0.86	0.95	<0.001
Severe	0.90	0.89	0.91	<0.001	0.85	0.81	0.90	<0.001
Very severe	0.87	0.87	0.88	<0.001	0.87	0.83	0.92	<0.001
**Respiratory Diseases**								
Mild	0.91	0.91	0.92	<0.001	0.90	0.86	0.94	<0.001
Moderate	0.92	0.91	0.93	<0.001	0.89	0.85	0.93	<0.001
Severe	0.87	0.87	0.88	<0.001	0.85	0.81	0.89	<0.001
Very severe	0.83	0.83	0.84	<0.001	0.84	0.80	0.88	<0.001
**Respiratory Failure**								
Mild	0.91	0.90	0.92	<0.001	0.93	0.86	1.01	0.105
Moderate	0.93	0.92	0.94	<0.001	0.92	0.86	0.99	0.031
Severe	0.86	0.85	0.87	<0.001	0.88	0.81	0.95	0.002
Very severe	0.84	0.83	0.85	<0.001	0.88	0.81	0.96	0.004

Note: CI, confidence interval. All models were analyzed via the generalized estimating equation. Extraneous factors adjusted in the model contained type of disability, age, sex, premium salary, urbanization level, CCI score, catastrophic illness status, long-term care facility residents, outpatient utilization, hospital admission, and preventive care service utilization.

## Supplementary Materials

The following are available online at https://www.mdpi.com/2076-393X/8/1/140/s1, Table S1: Baseline characteristics of elderly individuals with disability before and after propensity score matching.
