# Supplementary material for: Protective Effect of Seasonal Influenza Vaccination in Elderly Individuals with Disability in Taiwan: A Propensity Score–Matched, Nationwide, Population-Based Cohort Study
Source: Vaccines (Basel). 2020 Mar 22;8(1):140. doi: 10.3390/vaccines8010140 (PMC7157623; doi:10.3390/vaccines8010140)
Supplement: Supplementary file 1 [file vaccines-08-00140-s001.pdf]

vaccines

MDPI

Supplementary Material

Supplementary Table 1. Baseline characteristics of elderly individuals with disability before and after propensity score matching.

Variables	Crude Data							After Matching						
	Total		Without IV		With IV		*p*-value [1]	Total		Without IV		With IV		*p*-Value [1]
	N	%	N	%	N	%		N	%	N	%	N	%	
**Total**	394,490	100.00	258,042	100.00	136,448	100.00		272,896	100.00	136,448	100.00	136,448	100.00	
**Disability severity**							<0.001							<0.001
Very severe	50,672	12.84	32,087	12.43	18,585	13.62		36,240	13.28	17,655	12.94	18,585	13.62	
Severe	67,426	17.09	44,291	17.16	23,135	16.96		46,714	17.12	23,579	17.28	23,135	16.96	
Moderate	122,847	31.14	82,168	31.84	40,679	29.81		83,828	30.72	43,149	31.62	40 679	29.81	
Mild	153,545	38.92	99,496	38.56	54,049	39.61		106,114	38.88	52,065	38.16	54,049	39.61	
**Category of disability**							<0.001							<0.001
Physical	137,178	34.77	92,476	35.84	44,702	32.76		92,963	34.07	48,261	35.37	44,702	32.76	
Hearing	75,699	19.19	48,405	18.76	27,294	20.00		53,131	19.47	25,837	18.94	27,294	20.00	
Failure of vital organs	54,136	13.72	34,728	13.46	19,408	14.22		38,400	14.07	18,992	13.92	19,408	14.22	
Multiple	43,172	10.94	27,532	10.67	15,640	11.46		30,752	11.27	15,112	11.08	15,640	11.46	
Dementia	31,840	8.07	19,775	7.66	12,065	8.84		23,529	8.62	11,464	8.40	12,065	8.84	
Vision	27,856	7.06	18,450	7.15	9406	6.89		18,812	6.89	9406	6.89	9406	6.89	
Psychological	13,322	3.38	8885	3.44	4437	3.25		8049	2.95	3612	2.65	4437	3.25	
Other	11,287	2.86	7791	3.02	3496	2.56		7260	2.66	3764	2.76	3496	2.56	
**Sex** [2]							<0.001							0.638
Female	198,020	50.20	131,042	50.78	66,978	49.09		134,079	49.13	67,101	49.18	66,978	49.09	
Male	196,470	49.80	127,000	49.22	69,470	50.91		138,817	50.87	69,347	50.82	69,470	50.91	
**Age** (year) (mean ± SD) [2]	77.15 ± 7.88		76.76 ± 8.01		77.90 ± 7.56		<0.001	77.94 ± 7.67		77.99 ± 7.78		77.90 ± 7.56		1.000
65-69	80,224	20.34	58,494	22.67	21,730	15.93		43,460	15.93	21,730	15.93	21,730	15.93	
70-74	81,779	20.73	54,781	21.23	26,998	19.79		53,994	19.79	26,996	19.78	26,998	19.79	
75-79	80,924	20.51	50,615	19.62	30,309	22.21		60,615	22.21	30,306	22.21	30,309	22.21	
80	151,563	38.42	94,152	36.49	57,411	42.08		114,827	42.08	57,416	42.08	57,411	42.08	
**Premium salary** (NTD) [2]							<0.001							0.996
<19 273	133,071	33.73	87,430	33.88	45,641	33.45		91,231	33.43	45,590	33.41	45,641	33.45	
19 274-22 800	161,451	40.93	102,183	39.60	59,268	43.44		118,594	43.46	59,326	43.48	59,268	43.44	
22 801-45 800	61,481	15.58	42,352	16.41	19 129	14.02		38,256	14.02	19,127	14.02	19,129	14.02	

45 801	38,487	9.76	26,077	10.11	12,410	9.10		24,815	9.09	12,405	9.09	12,410	9.10	
**Urbanization level** [2]							<0.001							0.835
Urban	191,671	48.59	131,220	50.85	60,451	44.30		120,902	44.30	60,451	44.30	60,451	44.30	
Suburban	134,518	34.10	85,329	33.07	49,189	36.05		98,266	36.01	49,077	35.97	49,189	36.05	
Rural	68,301	17.31	41,493	16.08	26,808	19.65		53,728	19.69	26,920	19.73	26,808	19.65	
**CCI score** [2]							<0.001							0.986
0	77,760	19.71	57,076	22.12	20,684	15.16		41,368	15.16	20,684	15.16	20,684	15.16	
1-2	160,317	40.64	104,604	40.54	55,713	40.83		111,426	40.83	55,713	40.83	55,713	40.83	
3-4	96,414	24.44	59,497	23.06	36,917	27.06		73,898	27.08	36,981	27.10	36,917	27.06	
5	59,999	15.21	36,865	14.29	23,134	16.95		46,204	16.93	23,070	16.91	23,134	16.95	
**Catastrophic illness**							<0.001							0.109
No	310,708	78.76	204,130	79.11	106,578	78.11		212,809	77.98	106,231	77.85	106,578	78.11	
Yes	83,782	21.24	53,912	20.89	29,870	21.89		60,087	22.02	30,217	22.15	29,870	21.89	
**Long-term care facility residents**							<0.001							<0.001
No	375,171	95.10	250,366	97.03	124,805	91.47		256,503	93.99	131,698	96.52	124,805	91.47	
Yes	19,319	4.90	7676	2.97	11,643	8.53		16,393	6.01	4750	3.48	11,643	8.53	
**Outpatient utilization** [3]							<0.001							<0.001
<18	145,799	36.96	109,762	42.54	36,037	26.41		89,384	32.75	53,347	39.10	36,037	26.41	
≧18	248,691	63.04	148,280	57.46	100,411	73.59		183,512	67.25	83,101	60.90	100,411	73.59	
**Hospital admissio**n [3]							<0.001							<0.001
No	295,965	75.02	194,828	75.50	101,137	74.12		200,146	73.34	99,009	72.56	101,137	74.12	
Yes	98,525	24.98	63,214	24.50	35,311	25.88		72,750	26.66	37,439	27.44	35,311	25.88	
**Preventive care service utilization** [3]							<0.001							<0.001
No	304,258	77.13	212,516	82.36	91,742	67.24		203,357	74.52	111,615	81.80	91,742	67.24	
Yes	90,232	22.87	45,526	17.64	44,706	32.76		69,539	25.48	24 833	18.20	44,706	32.76	

Abbreviations: IV, influenza vaccine; CCI, Charlson Comorbidity Index; NTD, New Taiwan dollar. [1] Chi-square test. [2] Matching variables. [3] The utilization status during the preceding 9 months.
